# Women’s embodied experiences of second trimester medical abortion

**DOI:** 10.1177/0959353517692606

**Published:** 2017-01-01

**Authors:** Carrie Purcell, Audrey Brown, Catriona Melville, Lisa M McDaid

**Affiliations:** MRC/CSO Social and Public Health Sciences Unit, University of Glasgow, UK; NHS (National Health Service), UK; NHS Ayrshire and Arran, UK; MRC/CSO Social and Public Health Sciences Unit, University of Glasgow, UK

**Keywords:** second trimester abortion, embodiment, pregnancy, liminality, lived experience

## Abstract

Abortions in general, and second trimester abortions in particular, are experiences which in many contexts have limited sociocultural visibility. Research on second trimester abortion worldwide has focused on a range of associated factors including risks and acceptability of abortion methods, and characteristics and decision-making of women seeking the procedure. Scholarship to date has not adequately addressed the embodied physicality of second trimester abortion, from the perspective of women’s lived experiences, nor how these experiences might inform future framings of abortion. To progress understandings of women’s embodied experiences of second trimester abortion, we draw on the accounts of 18 women who had recently sought second trimester abortion in Scotland. We address four aspects of their experiences: later recognition of pregnancy; experiences of a second trimester pregnancy which ended in abortion; the “labour” of second trimester abortion; and the subsequent bodily transition. The paper has two key aims: Firstly, to make visible these experiences, and to consider how they relate to dominant sociocultural narratives of pregnancy; and secondly, to explore the concept of “liminality” as one means for interpreting them. Our findings contribute to informing future research, policy and practice around second trimester abortion. They highlight the need to maintain efforts to reduce silences around abortion and improve equity of access.

While an increasing proportion of abortions worldwide now take place early in pregnancy, around 10–15% of abortions take place at relatively later gestations (Gemzell-Danielsson & Lalitkumar, 2008). Women seeking *later second trimester abortion* – a phrase which we use in this paper to denote weeks 16–24 – in some settings continue to represent a relatively small but consistent number of women terminating their pregnancies (Guttmacher Institute, 2014). For example, Scottish data have since 2005 put the proportion treated at 14 weeks or more at 5% of all abortions (Information Services Division, 2016).

Research on second trimester abortion has tended to focus on the relative risks of medical and surgical methods (Lohr, Hayes, & Gemzell-Danielsson, 2008) and the characteristics and decision-making of women seeking it (Jones & Finer, 2012; Lee & Ingham, 2010). The psychologisation of abortion – particularly attempts to establish so-called “post-abortion syndrome” as a diagnostic category – has been addressed across gestations, and critiqued for taking insufficient account of contextual factors contributing to women’s decisions and experiences around abortion (Macleod, 2012). Little research has been grounded in lived, embodied experiences of second trimester abortion, as a means of addressing how women experience what happens in/to their bodies at this time. This knowledge gap has significance regarding the broadening of abortion provision worldwide, and ongoing attempts to undermine access to safe, legal abortion where currently provided.

Second trimester abortion tends to be framed as a “problem” for public health and for women since it is more costly and more medically risky than earlier abortion procedures – although, where safely provided, abortion (including second trimester abortion) remains safer than childbirth (Janiak, Kawachi, Goldberg, & Gottlieb, 2014). Where abortion is illegal or not publicly funded, practical and financial barriers are considerable, and increase with gestational age (Bloomer & O’Dowd, 2014; Janiak et al., 2014; Lince-Deroche et al., 2015). In practice, gestation significantly impacts provision and, even where second trimester abortion is legally available, significant barriers may exist, such as the need to travel substantial distances for treatment (Doran & Hornibrook, 2014; Purcell, Cameron, et al., 2014; Sethna & Doull, 2013). Evidence suggests that women have greater “reservations” around second trimester procedures than first trimester abortion (Lie, Robson, & May, 2008) and are more likely to continue with a pregnancy when presenting later at services (Cameron et al., 2016).

Abortion continues to occupy an ambiguous sociocultural position in that, in many contexts where it is legal, it is not constructed solely as a healthcare procedure. In Britain (i.e. Scotland, Wales and England, excluding Northern Ireland), abortion is provided by the National Health Service to 24 weeks, but technically remains a criminal offence outside specific legal grounds, and must be deemed necessary in every instance by two physicians (Abortion Act, 1967; British Pregnancy Advisory Service, 2013). This legislation extends to Scotland, where our research took place, although powers over this law were in 2016 transferred to the devolved Scottish Parliament (which has since 1997 controlled all other aspects of healthcare provision). The 1967 Abortion Act does not extend to Northern Ireland where, despite recent attempts to challenge the law, abortion is permitted only in the rarest circumstances, and is governed by a law dating from 1861.

Although it is a lived reality for approximately one in three women of reproductive age (Stone & Ingham, 2011), abortion continues to be framed as a topic for abstract public debate, with a common focus on gestational time limits (Purcell, Cameron, et al., 2014). Second trimester abortion is thus especially problematic, since it is an acutely contested area of an already stigmatised practice (Norris et al., 2011). Moreover, gestation impacts women’s access to abortion in Scotland. While legally permissible, an absence of provision beyond 18–20 weeks means women seeking abortion for non-medical indications (Ground C of the 1967 Abortion Act) are required to travel to England for the procedure (see Purcell, Cameron, et al., 2014).

Women in Scotland undergoing second trimester abortion (for both medical and non-medical indications) up to 18–20 weeks are typically offered a procedure using medication (i.e. “medical”, as distinct from “surgical” abortion). Here the woman is conscious and passes the fetus vaginally, under nursing supervision in hospital. The later medical abortion process can be physically challenging and potentially distressing, since women typically experience pain and vaginal bleeding accompanied by nausea, vomiting, fever and diarrhoea (medication side-effects) lasting four to 24 hours, until the fetus is expelled. In contrast to *earlier* medical abortion, second trimester medical abortion can resemble an induced labour, since the woman has to actively “push” to expel the fetus, although in many cases this would be passed on a bedpan or commode, rather than supine on a hospital bed.

From around 20 up to 24 weeks, women terminating on non-medical grounds are required to travel to specialist clinics in England, usually for a surgical procedure. Surgical second trimester abortion, which is not currently provided in Scotland, involves a two-stage “dilation and evacuation” process under general anaesthesia, where the fetal heartbeat is stopped, the cervix softened, and the pregnancy tissue removed by a doctor using forceps and suction. Whereas women undergoing later abortion for non-medical indications are managed through NHS Scotland hospital gynaecology services, women terminating for medical indication (such as fetal anomaly) are managed via obstetrics services, are treated using medical methods, and are not required to travel to England.

Existing research on women’s experiences of abortion highlights constraints which dominant social narratives place on women’s ability to account for their experiences of ending a pregnancy, and the absence of “pro-choice language for talking about the nitty-gritty of abortion” (Simonds, Ellertson, Springer, & Winikoff, 1998, p. 1317). Difficulties are confounded – and public narratives further muddied – by anti-abortion groups’ deployment of images of second or third trimester fetuses to represent the products of conception expelled in earlier procedures (Kelland & Macleod, 2015). Since silence and ambiguity tend to prevail around abortion in many cultural contexts worldwide, women have few opportunities in everyday life for safe and frank discussion of what abortion involves and feels like. There are both advantages and risks associated with increased public discussion of abortion, as highlighted by Stephenson, Mills, and McLeod (2017), including that frank discussion of the details of abortion provision may feed into attempts to restrict access. We argue, however, that calls for greater openness amongst providers regarding second trimester provision (Harris, 2008) would be complemented by greater sociocultural visibility of women’s experiences. Greater visibility has the potential to improve understandings of the ways in which women’s experiences are framed and constrained by dominant narratives of pregnancy and abortion. Insight here can inform future research, policy and practice, and highlight areas most in need of work to challenge negative framings and improve access.

To contribute to this understanding, we draw on data from a qualitative study of women seeking later second trimester abortion (in this case at ≥16 weeks’ gestation) conducted in Scotland in 2013. In analysing these data, it became clear that women’s accounts were strongly grounded in their embodied experiences of four aspects of pregnancy and abortion, each of which are examined in this paper. These related to: later recognition and confirmation of pregnancy; experiencing a pregnancy of relatively advanced gestation which ended in abortion; experiences of the abortion procedure; and the subsequent bodily transition. Each component does not necessarily feature in the experience of all women seeking second trimester abortion. However, as potential constituent parts of the phenomenon, they converge to constitute an experience which is socially ambiguous, taboo, and commonly positioned as being at odds with dominant narratives of pregnancy and feminine embodiment.

To contextualise our analysis, we explore key contributions in the existing literature on second trimester abortion and pregnant embodiment. We address how existing research suggests we might understand women’s experiences around second trimester abortion, and highlight gaps in this knowledge. Given the dearth of literature on embodied experiences of abortion, we draw on scholarship on a range of pregnancy experiences, including that which has previously applied the anthropological concept of “liminality” to pregnancy. We then present our findings and discuss their implications in this context, and their potential to inform abortion policy, provision and support for women seeking second trimester abortion.

## Problematising embodied experiences of second trimester abortion

### Understanding second trimester abortion

Relatively little scholarship has addressed women’s embodied experiences of medical abortion, aside from a key contribution from research conducted when these methods first emerged and were not yet established practice (Simonds et al., 1998). Simonds et al.’s research addresses only early gestations, but raises a number of points of contrast between surgical and medical methods, specifically: that women tended to align the early medical process with miscarriage, which enabled them to frame it as relatively “natural”; the likelihood for medical abortion to create a more immediate encounter between women and the aborted embryo/fetus; and the woman’s relatively more active role in the abortion process. The ways in which these issues play out in second trimester abortion are explored below.

Beyond this contribution, existing research on women’s experiences of abortion have tended to focus largely on context, reasons/decision-making, and stigma (Purcell, 2015). While we address these factors elsewhere (Purcell, Cameron, et al., 2014), abortion stigma has some relevance to the present context, since it is sociocultural context (specifically the social significance attributed to women’s pregnant bodies and to the embryo/fetus), rather than any inherently negative characteristic, which impacts negatively on women who undergo abortion (Hanschmidt, Linde, Hilbert, Riedel-Heller, & Kersting, 2016; Kimport, Foster, & Weitz, 2011; Norris et al., 2011). Stigma arguably also prevents the normalisation of abortion by obscuring it, creating a “prevalence paradox” in which exceptionality is assumed due to infrequent disclosure (Kumar, Hessini, & Mitchell, 2009). However, a critique of the recent scholarly focus on abortion stigma suggests that an exclusive focus on stigma elides more fundamental structural issues and inequalities, and calls for alternative approaches to understanding how and why the ambiguous position of abortion is maintained (Kumar, 2013). As Katz (2017) highlights in her problematisation of the so-called “pro-voice” approach to abortion counselling, it is crucial that abortion scholarship and advocacy addresses not only women’s lived experiences, but also the structural inequities which frame and constrain those experiences.

Developments in abortion provision – particularly the now widespread use of medical methods – have resulted in an increasing proportion of abortions carried out at earlier gestations in contexts such as Britain. However, a small but significant number of women continue to require abortions later in pregnancy, for a variety of complex and multi-factorial reasons including later recognition of pregnancy (Jones & Finer, 2012; Purcell, Cameron, et al., 2014).^1^ Factors that contribute to later recognition, and which often feature in accounts of second trimester abortion, include: not expecting to be pregnant; contraceptive use; ongoing bleeding taken for menstruation; absence of/not recognising signs of pregnancy; and avoiding confirmation due to ambivalence (Lee & Ingham, 2010; Purcell, Cameron, et al., 2014).

A potential factor in second trimester abortion, later-recognised pregnancies can be understood as problematic in various ways. They sit uncomfortably with normative western sociocultural narratives of feminine embodiment which construct women as essentially in tune with their bodies and in “control” of their fertility (Martin, 1992), primarily through active participation in contraceptive use (Hoggart & Newton, 2013). While a woman’s embodied experience of “quickening” (i.e. perception of fetal movement) was in the past taken as the key confirmation of pregnancy, modern technologies of pregnancy detection (including home testing and ultrasound) have normalised *early* identification of pregnancy. Narratives of how pregnancy experiences “should” be are pervasive, in popular culture, and in literature targeted at pregnant women (Marshall & Woolett, 2000). A normative narrative emerges from these sources, comprising: “a missed period, a home pregnancy test, and a medically managed pregnancy prominently featuring visits to view the developing ‘baby’ via ultrasound” (Peel & Cain, 2012, p. 79). Deviations from this template are less well-represented, with potentially alienating consequences for those who experience them.

Where broader cultural narratives address abortion, representations tend to be highly negative, sensationalist, and to exclude the voices of women who have experienced it (Evans & O’Brien, 2014; Purcell, Hilton, & McDaid, 2014). This is so even where public opinion is largely in favour of women’s right to abortion, as polling suggests is the case in Britain (Abortion Rights, 2015; Lee, 2000). The potential for negative sociocultural narratives – or the absence of any recognisable narrative at all – to shape expectations and experiences may be especially acute around the often publicly contested ground of later abortion. Moreover, while the internet offers potential for public sharing of abortion stories – for example via www.theabortiondiarypodcast.com or www.womenonweb.org – such platforms may not be widely known, of obvious relevance or, in resource-poor settings, accessible at all. The internet is also a significant source of misinformation on abortion, meaning accurate information and advice may be obfuscated (Rowlands, 2011). Where inaccuracies and silences prevail, abortion and other pregnancy experiences which do not result in a live baby continue to be understood as non-normative and exceptional, limiting possibilities for improved healthcare and social support.

### Pregnancy and liminality

The anthropological concept of “liminality” (derived from the Latin for “threshold”) describes the middle stage in a rite of passage when the subject is “betwixt and between” two social categories (Turner, 1964; Van Gennep, 2004[1909]). The beginning of this period is typically marked by a process of physical or discursive “separation”. Following this separation, those in a liminal state are rendered structurally – and in some cases physically – “invisible” for the duration of this period. The temporary state of liminality usually ends with a process (or ritual) of “re-assimilation”, in which those in the liminal state either progress forward to the next social category or, more problematically, return to their original pre-rite state. Liminality also bears strong association with concepts of pollution and “matter out of place” (Douglas, 1966). Insofar as liminal individuals do not clearly fit one category or another, they are typically viewed as symbolically polluting and threatening to the social order. This order is maintained in the enacting of associated taboos, which prohibit or constrain representations of the liminal being.

The potential relevance of liminality to pregnancy is twofold. Firstly, the embodied experience of pregnancy can be viewed as a rite of passage, which comprises a period of liminality, in the transitional physical and social state that it comprises (Balin, 1988, drawing on Van Gennep, 2004[1909]). This period of liminality is typically presented in the literature as beginning, with a pregnancy test, or with the social acknowledgment of a pregnancy. The normative expectation is that the transitional period will end in the ritual of childbirth, which comprises both a physiological and a social event. Within the range of women’s pregnancy experiences, however, a number of further components of liminality can be identified. These in part depend on where the threshold of pregnancy is understood to be located, and can be viewed as relating to the early stages of pregnancy and to its resolution. Before a pregnancy has been confirmed or publicly acknowledged, a woman may experience a sense of liminality in which she is “neither pregnant nor ‘unpregnant’” (Peacock et al., 2001). In this context, women express concerns about others’ perceptions of their bodies, in a way which highlights the significance of a pregnancy’s physical manifestation in the woman’s changing body shape (Nash, 2012; Neiterman, 2013; Nicolson, Fox, & Heffernan, 2010). The visibility of pregnancy to others becomes a significant factor in how women experience the pregnancy and others’ judgements of it (Nash, 2012). Aside from one existing contribution, which addresses the issue from a very different perspective (Boltanski, 2013 – see Lewis’s (2017) review of this book in Part 1 of this special issue), experiences of the early stages of a pregnancy that ended in abortion – and the significance of the physical and social visibility of the pregnancy – have not yet been explored.

At the other end of the pregnancy process, childbirth has been framed as a liminal process, in that the body of the woman and fetus are neither the same nor separate, in a way which can be both physically and existentially challenging for women (Lupton & Schmied, 2013). As that research suggests, the psychosocial impact of whether or not a woman is conscious at this moment of separation may be significant for how she experiences this event and adjusts to its aftermath. While different in their sociocultural location, the physical similarities between labour and second trimester medical abortion suggest it is worth exploring the implications of (un)consciousness for women undergoing this method.

Liminality is also mooted as a means of understanding miscarriage, which can be framed as occurring in an ambiguous sociocultural space between being and not being pregnant/a parent (Reiheld, 2015). Typically associated with taboo, misunderstanding and silence, the “betwixt and between” position generated by miscarriage relates not only to the woman, but also to the miscarried embryo/fetus. The latter may be regarded as both acutely liminal (Layne, 2003), and as “matter out of place” in the sense that they have separated from the woman’s body earlier than desired (Murphy & Philpin, 2010). Crucially, difficulties for women may be perpetuated by an absence of sociocultural scripts which adequately describe these experiences (Peacock et al., 2001; Reiheld, 2015). These points suggest that the liminality of pregnancy as an embodied state, and as a supposed transition between social categories, may also have relevance for how women experience a second trimester pregnancy which ends in abortion.

Liminality has previously been deployed to highlight the way in which abortion in general disrupts the normative progression of pregnancy as a rite of passage (Ginsburg, 1989), in that the transition does not end with the birth of a live baby, to which the woman is mother. However, it has not yet been used to specifically draw out women’s embodied experiences up to and around second trimester abortion. Each of the uses noted above, and the original conceptualisation of liminality, suggests its relevance to understanding women’s experiences up to and around second trimester abortion. In particular, addressing liminality in this context offers an alternative means of understanding women’s experiences around second trimester abortion, and has the potential to draw out not only issues of stigma, but also fundamental social-structural issues which contribute to maintaining the invisibility of this phenomenon.

## Methodology

The data presented here are from a study of women seeking abortion at ≥16 weeks’ gestation in Scotland in 2013. This exploratory study was devised to investigate women’s experiences of later presentation, abortion and travel (see Purcell, Cameron, et al., 2014). Women terminating for medical indications (e.g. fetal anomaly, risks to the woman’s life – which in 2014 made up 29% of post-14 week abortions) were excluded from the study, given the different contextual considerations and management by health services. Given the potential vulnerability of women in this position, we excluded those who: were aged under 16 years; sought abortion as a result of sexual coercion; had insufficient English to be interviewed without an interpreter; were unable to provide fully informed consent; or appeared to be overly distressed during the clinic attendance. Ethical approval for the study was granted by the West of Scotland Research Ethics Committee 4.

A total of 48 women initially agreed to take part, and we were then able to contact and interview 23 participants. This sample represents 18% of the 125 women who presented at ≥16 weeks in participating clinics in the recruitment period (January–July 2013). Five of these had decided to continue their pregnancy by the time of their interview – primarily due to its later gestation or to guarantees of social support (Purcell, Cameron, et al., 2014) – and are thus excluded here. Fourteen of those who proceeded had medical abortions, and four had surgical abortions in England. Five participants were single at the time of their interview and, of the 12 who were in a relationship, four were in a different relationship to the one in which they had conceived. Six of the 12 in relationships were cohabiting, including one who was married. Six participants were currently living in social housing, seven in owned or privately rented accommodation, and five were living with their parents. Ages ranged from 17 to 39, with a mean age of 25. Eleven participants had children, and two had previously undergone abortion. All 18 women described their pregnancies as unintended. Abortions had been carried out at between 16 and 22 completed weeks.

Women were recruited by specialist nurses when presenting for abortion, and interviewed two to four weeks after treatment. Interviews were loosely structured, and used a topic guide – informed by the literature and study research questions – which focused on reasons for requesting abortion, for presenting “later”, and experiences of the process. To offer as much control as possible to participants, interviews followed the lead of interviewees (Kvale & Brinkmann, 2009). Interviews ranged from 35 minutes to two hours in length, and were conducted in various locations (primarily participants’ homes), as per the participant’s preference.

Following initial coding used to explore emerging issues (Saldana, 2013), women’s embodied experiences emerged as a key theme. Focused coding then identified data relating to this key theme, and captured ways in which women’s accounts of unplanned/later-recognised pregnancies and subsequent second trimester abortions were grounded in their embodied experiences, expectations of their reproductive bodies, pregnancy norms, and larger-scale cultural narratives around pregnancy and abortion. The first author performed the coding and analysis in consultation with the last author.

## Results and analysis

The analysis presented here considers women’s accounts of the process leading to a second trimester abortion, including later discovery of pregnancy and experiences of a second trimester pregnancy which ended in abortion. It addresses experiences of the medical abortion procedure, how these contrast with later surgical methods, and accounts of returning to “normal” following second trimester abortion. Each aspect relates to women’s embodied experiences of the pregnancy and abortion process, and to the liminality of these, in different ways. Identifiers following verbatim quotes indicate pseudonym, age, and gestation at abortion.

### Later recognition of pregnancy

The physical and social ambiguity of the early stages of pregnancy were highlighted in our participants’ accounts of not having known they were pregnant. Twelve of the 18 women included here were ≥12 weeks pregnant when the pregnancy was confirmed by a doctor. For some, this was because they were using contraception, had recently had a baby, had irregular periods, or were otherwise not expecting to be pregnant. Participants explained that what might be considered the “typical” physical signs of pregnancy were disguised or absent and, for many, the most significant issue was that they had not felt or looked pregnant:

See how your boobs grow a wee bit? That didn’t happen. My hair didn’t get thicker, it didn’t do that glow thing. I wasn’t getting any morning sickness. There was *nothing* indicating that I was pregnant. I hadn’t even put on *weight*. […] I didn’t have a wee bump or anything like that. I didn’t *look* pregnant, you know? And I didn’t *feel* pregnant. (Tia, 20, 16 weeks)

Tia – who had an eight month old and had until recently been in homeless accommodation – focused on what *did not* happen, and exemplifies an expectation that clear physical signs and bodily changes would accompany pregnancy, and that women will recognise a “feeling” of being pregnant. Particularly for those who had been pregnant before and expected to recognise these “signs”, their absence meant that they did not suspect pregnancy at all, or until a relatively advanced gestation.

Those who had not suspected pregnancy at all expressed astonishment, confusion and dismay that they could be pregnant for so long without consciously recognising it. Participants were acutely sensitive to how a later discovery of pregnancy might appear to others. Emma, for example – who had previously experienced stress-related amenorrhea and had attributed the absence of her period this time to the same cause – repeated phrases like “it must sound crazy”, “it’s strange”, and “I know it sounds awful”. Emma’s experience was shaped by her living circumstances, since she was at the time living abroad in a country where second trimester abortion is prohibited, where she had a highly negative experience of health professionals (including the unbidden involvement in her medical care of members of the clergy), and where her partner had been unsupportive.

The feelings women expressed about not having fit the “typical” model of pregnancy discovery point to the physical, psychological and social significance of this moment, which can potentially be regarded as marking the threshold of pregnancy. For them, this discovery did not necessarily coincide with a public acknowledgement of pregnancy, since most kept the fact of the pregnancy to themselves and/or a close circle of others. However, the confirmation of the pregnancy did mark a significant shift in their self-perception. Like Emma, other participants imagined or expected that they would be negatively judged or perceived as stupid, and were keen to emphasise that they genuinely had not known nor had reason to suspect pregnancy:

I just kept thinking, “I can’t believe … I’ve not known …” Like, I felt really stupid that – how can you be, well, nearly five months pregnant, and not even know about it? […] So many things were kinda just disguising it that, I never once thought … ‘cause I’m not daft. I would go and get a pregnancy test! But, I didn’t even have any doubts. […] But, the thought of being, like, somebody on Jeremy Kyle, or something, you know? (Fiona, 28, 19 weeks)

Having initially home-tested, Fiona had expected to be only 3–4 weeks pregnant, and encapsulated the sense of disbelief and potential for negative judgement with her allusion to a tabloid-style television talk show.

### Experiences of second trimester pregnancy, where pregnancy ended in abortion

Women’s embodied experiences of being pregnant, when this had ended in second trimester abortion, were also varied and complex, and descriptions foregrounded the significance of women’s (changing) corporeality in this context, for themselves and others. Tia (quoted earlier) highlighted the significance of the absence of bodily signs which may have suggested pregnancy. However, these could be equally problematic where they did start to become evident, making the woman acutely conscious of bodily changes. Accounts of these issues focused particularly around the emerging pregnancy “bump”. This potential physical marker was experienced negatively by Fiona, who – as was the case with several women – stayed home from work after the sudden discovery that she was 18 weeks pregnant:

I knew that I didn’t want a baby. But, because I was showing it made it really difficult. […] I had to speak to my boss at my work. Luckily I was able to work from home, because everybody would’ve known, ‘cause I had an obvious bump. So, I kinda had to just hide myself away so nobody noticed. (Fiona, 28, 19 weeks)

Fiona described sequestering herself so that what she felt was now quite obvious physical evidence of her pregnancy would not be noticed by others, and she would not feel obliged to explain her situation. She felt this was particularly acute since the long-term relationship in which she had conceived had ended months before, meaning “everybody would know that I’ve- that it wasn’t *planned*”.

Unlike Fiona, Quinn was initially undecided about the pregnancy, in part due to her partner being originally pleased and then withdrawing support, prompting the relationship to break down (another common experience). A comment from her mother on her changing body shape highlighted for Quinn that she was on the threshold of the pregnancy being physically and socially visible, which crystallised her decision and prompted her to take action:

… everything was just going through my head. I was like “Mum I don’t think I can *do* this”. And of course my mum would *never* have said anything. She just listened. And when we got to [mum’s house] she said to me “oh look, I can see a bit of a tummy”, and I was like “can we just not *talk* about it please?” And I think that was the first time I went “right, I think I know what decision I’m making here”. (Quinn, 33, 16 weeks)

As well as whether or not they “felt” pregnant, the visibility of the pregnancy (as a signifier to others and themselves) was tied to understandings of the advancing nature of the pregnancy and fetal development, as were their accounts of feeling sensations of fetal movement. Beth, for example, had difficulty deciding whether to terminate, and her need to make a final decision hinged less on its external visibility and more on the significance she placed on fetal movement:

I was getting wee flutters and that. Not- nothing major, no big movements. But, y’know, I was getting wee flutters and all that so I was like: “I need to make my mind up because I can actually *feel* it.” (Beth, 31, 17 weeks)

Beth framed the latest point at which, for her, it would be acceptable to end the pregnancy as being the threshold at which fetal movement became more noticeable.

Not all such experiences were described negatively, however. Melissa, for example, had just left school, was seeking employment and, like Beth and three others, had initially considered continuing the pregnancy. On reflection, Melissa decided that her partner was not a suitable parent (the relationship then ended) and she did not want to rely on welfare support. She spoke in relatively positive terms of the bodily transition she experienced before her decision to terminate:

My bump was so big, like, I used to always touch it. I was catching myself putting my hand on my stomach […]and, like, when it would kick I would be like: “oh my god!” I’d freak out. *(CP: Right, so you could feel movement and stuff then?)* Yeah, so much, it was so weird. I used to love it, I used to think it was amazing, just thinking there was a life inside me, it was lovely. (Melissa, 17, 18 weeks)

Melissa’s account was atypical for this framing of fetal movement and the “bump” and, while described positively, this was nevertheless a private experience and not something she had felt able to share with others.

Some women who had initially considered continuing the pregnancy described engaging with what might be viewed as the pregnancy-related “rituals” – for example taking folic acid supplements and eating certain foods – as a means of countering any potential damage that might have been done before they knew they were pregnant:

The first week [*after discovering pregnancy*] I was convinced I was having a baby, so I was taking like folic acid tablets, I started eating all the stuff you're supposed to eat. I was feeling awful, because … […] I was like “oh god, there was like this night where I'd had a couple of glasses of wine with a meal”. And then I was thinking “well, I have a coffee every day” […] and I thought “oh god, I've been doing that and I didn't know up until now”. So then I cut out those things. (Emma, 21, 18 weeks)

Even those who had fairly swiftly decided on abortion alluded to the expectation that they would engage in the self-regulating practices expected of pregnant women (such as avoiding alcohol, smoking, and certain medications). Vivienne (19, 19 weeks), for example, noted: “Being on like my antidepressants I thought there might have been something wrong with the baby, and, em … I’d been out drinking and things as well.” For women who raised this issue, it was presented as supplementing their decision to terminate, in relation to concerns that damage may have been done to the fetus.

### The “labour” of second trimester abortion

As well as the impact of later discovery, and their ambiguous position following discovery and before abortion, women’s accounts of the second trimester medical abortion procedure describe a highly ambiguous experience, which was at times difficult to articulate. This was primarily so around key similarities and differences between this process and childbirth. Many referred to (and recounted health professionals as describing) the second trimester medical abortion procedure as a “mini labour” or “giving birth”, highlighting parallels with childbirth in the way second trimester abortion is narratively constituted. The majority of participants described not having anticipated how closely the process might resemble experiences of childbearing, however. For example, Quinn said “I just don’t think I was prepared for … the labour […] That was like ‘whoa, we are actually gonna do this’”; while Tia noted: “it did feel like labour all over again, without the drugs”. Fiona explained:

I thought that it just sorta would happen, I never realised that it was gonna be like actually giving birth, and having to- like, I got contractions and had to *push* […] but I didn’t realise I’d have to go through what you do for actually … So, that made it really … I think that made it a lot *worse*, ‘cause like I’m doing all this, and then at the end of it you’re … and … So, that was horrible. (Fiona, 28, 19 weeks)

Fiona’s hesitant description exemplifies the way in which many women struggled to articulate this experience.

Some participants suggested that they did not feel entitled to behave or be treated by health professionals in the same way as a woman in childbirth, and that there was a punitive element to this:

The doctor at one point said to me “what I’m gonna do is going to be quite painful […] Do you want some drugs or gas and air?” And it came off the tip of my tongue and I went “I don’t deserve it, so no”. […] I was beating myself up completely. (Paula, 37, 17 weeks)

For many participants, passing the fetus was distressing and painful. Beth described encountering the aborted fetus as the nurse removed it from the commode:

She had one of the … the pads that they put on the bed? Aye, she had one of them and the baby was on top of it, and she just put another one on top of the baby. But I … I’d seen … She was like “don’t look”, but as soon as somebody tells you not to look … But, I’d seen it anyway before she had said “don’t look,” so … Aye, it was horrible. (Beth, 31, 19 weeks)

As was the case with many participants, Beth went on to note that seeing the fetus was for her “the worst bit” of the experience. Beth had to be transferred from the dedicated abortion day ward to the hospital labour ward when the former closed in the early evening. Her distress was compounded by her acute awareness that her room in the labour ward “was … ready for a baby … not to do … what I was doing”.

For some women, however, the hospital context offered a relatively useful set of narrative resources upon which they could draw to explain and make sense of the abortion. Quinn explained her experience of different stages of the process, and the difference which passing the pregnancy while upright on a commode, rather than supine on a bed, made to this experience:

… it became medical […] you go from inducing labour, to being in labour, to actually delivering in a toilet, to then going back to a medical procedure and you’re stepping away from it every time. You’re getting further away from the baby side of things. […] Actually, that was a godsend, because I think had I been lying back in a bed and delivering, it would have just been like having [child] again and you’d have remembered it. Whereas being in a bathroom, sitting on a commode, you don’t associate that with labour. I associated it with almost just a medical “this is something we need to do”. (Quinn, 33, 16 weeks)

In this way her interaction with the medical environment helped Quinn to pin down and frame as a necessary healthcare procedure what, for many, was highly ambiguous and difficult to verbalise.

Several women described feeling “empty” following the abortion: both in a negative sense that they felt sadness at the situation, and in a more positive way, in that they no longer felt pregnant. Most (Beth included) described feeling powerful physical and emotional relief immediately on passing the fetus, since both the physical discomfort and the associated anxiety ended:

… when, like, as soon as it, like, *passed* I think I just felt so *relieved*, but like, because of all the pain I was in as well. It just stopped straight away. It’s like just, like, as soon as it … passed, that was it. (Jenna, 17, 17 weeks)

Women’s accounts of medical second trimester procedures contrasted notably with the four participants who had undergone surgical abortion under general anaesthetic in an English clinic (see also Purcell, Cameron, et al., 2014). For these women, accounts of the procedure were less extensive, focused chiefly on peri-operative care, and described a starker shift from pregnant to “unpregnant”:

I’d say that procedure’s much better than what they were gonna do in [Scotland], *much* better. ‘Cause you don’t- you’re sleeping. […] I was only sleeping for half an hour*. (CP: So, like, better in what way?)* ‘Cause you’re not seeing anything. Like, before they were wanting me to give birth to it. You’re not seeing anything, you’re not being- you’re not doing anything. […] You don’t know what’s going on, and when you wake up that’s it … really. (Natalie, 22, 20 weeks)

The accounts of women who had undergone surgical procedures thus appeared to be more distanced from the corporeality of the procedure, and particularly from any visceral encounter with the fetus. This served to further underline the vivid prominence of the later medical abortion process in the accounts of the majority who had undergone it.

### The transition back to “normal”

Some bodily changes were immediate following the abortion procedure, and for the most part participants regarded the pregnancy as having ended by the time they left the hospital or clinic. However, speaking two to four weeks after the abortion, most women also described another period of transition in which their bodies (and hence their day-to-day lives) returned to “normal”. This included several weeks of mood fluctuations, bleeding and other bodily processes. The former were not overly problematic since women were accustomed to managing bleeding and mood changes associated with their menstrual cycle. Others experienced lactation, however, which was unexpected and frightening:

I literally felt like I’d had [breast] implants put into me, and I got all worried. I had a look and my bra- it was just like covered in white stuff, and I was like, “oh my god …” and it was really sore as well. I was petrified ‘cause I didn’t get told that that would happen either. [Friend] was like: “don’t worry, that’s just your body getting rid of all the fluids, all the milk and stuff”, and I was like “ok … ?”. (Diana, 18, 18 weeks)

Even for those who had previously had children, this was very unsettling:

I actually started lactating again, which felt really strange, ‘cause my body kinda felt like it should have a baby, but my mind was like “no, you shouldn’t”. […] And after that I did get a wee bit emotional. […] After I’d come to grips with that I was just, like, you know what, I’m just gonna have to ride it out. (Tia, 20, 16 weeks)

Tia’s explanation puts her mind and body at odds in a way which expresses the complex combination of feelings many women had at this stage, and highlights a sense of embodied ambiguity that persisted with these experiences. Tia’s last sentence here also exemplifies the resoluteness most women expressed that once the physical symptoms had abated, they would be ready and able to move on.

## Discussion

The analysis presented above highlights challenges faced by women seeking second trimester abortion, grounded in their lived, embodied experiences. Our findings foreground elements of this experience which are socially ambiguous and taboo. It also demonstrates ways in which convergences of factors – including encounters with the aborted fetus, and being pregnant into the second trimester where the pregnancy ends in abortion – constitute challenges for women in this context. We now focus in more detail on the implications of these findings and what a conceptual perspective which focuses on issues of embodiment and liminality can add to understandings of second trimester abortion.

Firstly, we are able to address a number of issues relating to women’s embodied experiences up to and around second trimester abortion. The relationship between abortion and dominant sociocultural norms of feminine embodiment (including tropes of “nurturing motherhood” and “sexual purity”) renders the former problematic on multiple fronts (Norris et al., 2011). Our participants’ experiences suggest that second trimester abortion in particular conflicts with such norms, which essentialise women as having “intuitive” knowledge of their bodies (Martin, 1992). They are also at odds with normative scripts for the progression of a pregnancy. Participants’ emphatic shock at the later discovery of their pregnancies reflects the normalisation of early and straightforward discovery. While later discovery of pregnancy may in fact be reasonably common (Lee & Ingham, 2010; Peacock et al., 2001), our findings suggest that the dominant norm situates this as highly exceptional. Moreover, our findings indicate the impact which such positioning can have on women’s self-concept and experiences of their bodies. Women in this position faced coping with an unintended pregnancy *and* with the fact that they had been unknowingly pregnant for some time. The accounts of our participants point to these being unsettling corporeal experiences individually, and all the more so in combination.

Perceived fetal movement also appeared as a significant factor in participants’ experiences in and around second trimester abortion. In legislative terms, implantation and “viability” bookend the period in which abortion is possible (Sheldon, 2015). However, that fetal movement was noted as a significant point in pregnancy highlights the relevance of women’s embodied experiences to their attitudes towards their second trimester abortion. It suggests that, other sociocultural markers of the beginning of pregnancy aside, fetal movement continues to constitute a significant threshold, beyond which women may have more reservations about ending a pregnancy.

Visibility of the pregnancy signified the advancing pregnancy for women themselves. But perhaps more significantly, it was a potential marker of the pregnancy for others. While the pregnancy was physically invisible, many women preferred not to disclose it to others. Faced with having to act counter to powerful sociocultural norms of feminine embodiment, some women dealt with the liminality of this position by sequestering themselves, thus maintaining the pregnancy’s social invisibility.

Our results also point to the bodily transition which follows second trimester abortion as likewise presenting an unsettling bodily experience. This is not necessarily exclusive to second trimester abortion, and bodily transitions also feature in women’s accounts of childbirth (Lupton & Schmied, 2013). A key difference here is that, post-childbirth, women typically have more readily available sources of information, through friends, family, healthcare professionals or advice texts (Marshall & Woolett, 2000). As has been noted elsewhere, advice texts in particular function as “repositories of cultural ideas” (Oakley, 2016). Insofar as abortion is missing from these, it is also missing from any broader sociocultural narratives about pregnancy which might legitimise women’s experiences of second trimester abortion. Our findings suggest that women may instead be obliged to draw on those normative pregnancy narratives which are available, such as that of the “happy” pregnancy (Layne, 2003). This might include the expectation that women will be pleased on discovering their pregnancy, and that the outcome will be a healthy and wanted baby. As with miscarriage and stillbirth, the stark incongruences between this narrative and the lived experiences of women seeking second trimester abortion may exacerbate any difficulties experienced.

A lack of readily available narratives specifically of the second trimester medical abortion *procedure,* including how the fetus is actually expelled from the woman’s body, means that women undergo a largely unknown, unexpected and challenging process. This combination may leave them at a loss for how to make sense of this bodily experience. In this respect, our analysis suggests that women to some degree draw on narratives of childbirth in articulating the process, allusions which mark a clear divergence from accounts of women undergoing earlier medical abortion (Simonds et al., 1998). The latter have been found to align their experience with miscarriage, which had the parallel effect of “naturalising” the abortion. While the cultural visibility of narratives of childbirth (and perhaps increasingly of miscarriage) may mean that these are the most readily available for women to draw on, they may also compound negative feelings around this bodily experience in an extremely alienating way. Moreover, where our participants did align their experiences with childbirth, this was only partial, reflecting an experience which was in some respects like childbirth, or miscarriage, but nevertheless something ambiguous and in-between.

Drawing parallels between second trimester abortion and childbirth highlights a further point. The impact of the intense physicality of childbirth has been foregrounded (Lupton & Schmeid, 2013), particularly regarding the significance of whether the woman is under general anaesthetic, or awake and actively involved (for example in “pushing”). Our findings suggest a similar significance in the case of second trimester abortion, although further research would be required to explicitly compare experiences of medical and surgical procedures. What is evident from our findings is that an immediate encounter with a second trimester fetus can be highly distressing. A number of factors may contribute to this, not least the status of the fetus, which may be regarded as “matter out of place” (Murphy & Philpin 2010) – and thus a source of revulsion – or as “superliminal” (Layne, 2003), in the sense that it is an ambiguous product of an ambiguous process.

From these embodied experiences, a series of further factors emerge relating to the liminality of experiences up to and around second trimester abortion. Our findings suggest that factors which frame second trimester abortion not in terms of childbirth, but as an explicitly *medical* procedure, are perceived positively by some women, since its ambiguity is reframed in more definitive terms. There is a key tension to note here, in relation to established critiques of the medicalisation of women’s reproductive lives (Crossley, 2007; Oakley, 2016), and with aspects of the demedicalising potential that abortion medication may offer at earlier gestations (Simonds et al., 1998). We do not dispute the negative impact of the over-codifying of women’s reproductive experiences in medical terms, and that doing so can limit the possibility for alternative narratives. In the sense of offering women increased control and access, the demedicalisation of abortion is to be strongly welcomed. At the same time, the medical framing of the second trimester abortion procedure offers one means by which women that need to undergo it can more clearly conceptualise and legitimise their experience.

As it stands, however, the sociocultural positioning of abortion, in Britain and elsewhere, locates it as not exclusively a healthcare practice, but also a legal, moral and ethical issue. In countries where it is legalised, the very way in which it is legislated for maintains this ambiguous position (Boltanski, 2013). Moreover, second trimester abortion in particular tends to be presented, at the level of broad sociocultural narratives, as exceptionally negative and contested (Norris et al., 2011; Purcell, Cameron, et al., 2014; Purcell, Hilton, et al., 2014). This arguably contributes to the liminality of its lived experience, as women are left unclear as to whether their abortion is a medical procedure, something akin to the (socioculturally privileged) process of childbirth, or something else altogether. In the sense that it is an unwanted bodily state, pregnancy is arguably experienced by some women *as an illness*, and they seek to have their “normal” embodiment restored via medical means. From this perspective, clearer framing of second trimester abortion as an essential component of women’s healthcare provision offers advantages to women. It also offers a means of countering diversionary “moral” framings, such as those deployed by anti-abortion groups. While the full implications of this issue are beyond the scope of the present paper, it would be useful for further research to explore representations which are acceptable and relevant to the lived experiences of women in this position.

As with miscarriage, abortion may be viewed as rendering incomplete the “rite of passage” which pregnancy represents (Ginsburg, 1989; Layne, 2003). In the specific context of second trimester abortion, however, our findings suggest that women find themselves in what might be better described as a “superliminal” position not only “betwixt and between” the social categories of non-parent and parent, but also somewhere between “pregnant” and “unpregnant”. This was evidenced in concerns expressed about the (physical and social) visibility of their pregnancy. It was also evident in the transitional period after the abortion, where bodily experiences that might be expected to follow childbirth were unexpected and startling. The liminality of this position renders it structurally invisible (Turner, 1964) and subject to powerful taboos that prevent the construction of sociocultural narratives upon which women can draw to explain and make sense of their experiences. Although outside the remit of the present paper, it is interesting to note that the status of abortion as a private, individual experience seems to prohibit the “communitas” which would typically accompany a period of liminality, and in which women in the same position might exchange information and support. Further interrogation of this point would contribute to understandings of the liminality of both pregnancy and abortion.

As our analysis has demonstrated, there are a number of issues which addressing second trimester abortion in terms of embodiment and liminality illuminates, that thinking in terms of stigma alone would not. Ultimately, what this analysis suggests is that it is not only the challenges to norms of feminine embodiment posed by factors up to and around second trimester which contribute to silences around it. These silences also stem from the taboos which accompany – and act as a mechanism of control over – a liminal period or state which threatens a given social order. Addressing abortion in terms of liminality requires further theoretical work beyond the scope of this paper. What is clear, however, is that addressing abortion in these terms offers an alternative to the more commonly applied concept of stigma, which not only speaks to fundamental questions of social structure, but also is grounded in women’s lived, embodied experiences.

## Conclusions

The analysis in this paper highlights the impact which the absence of positive sociocultural scripts around second trimester abortion can have on women. These include later recognition of pregnancy, experiences of second trimester pregnancy which ends in abortion, the procedure itself, and the bodily transition which follows. Factors which could contribute to these include: the provision of visible, accurate information about what second trimester abortion involves; improving understandings of the range of experiences which might necessitate it; and the range of feelings women might experience around it. Greater discursive clarity around abortion, in which it is presented as a legitimate pregnancy outcome and reproductive healthcare procedure – and, in the case of Scotland, local provision of second trimester abortion up to the legal gestational limit – would also contribute to situating it in a clearer, socially valid position. There is also a clear need to further ascertain with what language women undergoing second trimester abortion feel comfortable, and to consider if and how alternative scripts can be claimed or established. Providing recognisable, visible and positive sociocultural narratives relating to second trimester abortion may be challenging, particularly vis-à-vis the vocal anti-abortion minority. However, silence also perpetuates its structural and social invisibility as a pregnancy experience. Despite the challenging experiences faced up to and around second trimester abortion, women nevertheless seek and proceed with treatment. There is therefore a need to ensure that policy makers and service providers are sensitive to these challenges, so that women seeking second trimester abortion are effectively supported.

## Note

1 While a diagnosis of severe fetal anomaly is another notable reason for second trimester abortion, the present paper addresses abortion for non-medical indications only (see section “Methodology”).
